# Retraction: Prospects and applications of nanobiotechnology: a medical perspective

**DOI:** 10.1186/1477-3155-10-40

**Published:** 2012-10-04

**Authors:** Md Fakruddin, Zakir Hossain, Hafsa Afroz

**Affiliations:** 1 Institute of Food Science and Technology (IFST), Bangladesh Council of Scientific and Industrial Research (BCSIR), Dhaka, Bangladesh; 2 Department of Microbiology, Primeasia University, Dhaka, Bangladesh

## 

This article [1] is retracted as it contains large amount of text that has been duplicated from other articles previously published. We apologize to all affected parties for the inconvenience caused.
